# Characterization of the complete mitochondrial genome of lesser grain borer *Rhyzopertha dominica* Fabricius (Insecta: Coleoptera: Bostrichidae) from Jingziguan

**DOI:** 10.1080/23802359.2019.1688120

**Published:** 2019-11-12

**Authors:** Bocheng Ouyang, Yu Bai, Jun Chen, Junyang Wang, Sheng Liang

**Affiliations:** a College of Mathematics and Information Science, Guiyang University, Guiyang, China;; b Guizhou Provincial Key Laboratory for Rare Animal and Economic Insects of the Mountainous Region, Guiyang University, Guiyang, China;; c School of Electronic and Communication Engineering, Guiyang University, Guiyang, China;; d College of Technology and Engineering, Beijing Normal University, Zhuhai, Zhuhai, China

**Keywords:** *Rhyzopertha dominica*, Bostrichidae, the lesser grain borer, mitochondrial genome, stored-product insect

## Abstract

The lesser grain borer, *Rhyzopertha dominica* (Fabricius) is a primary pest of starch-containing stored products worldwide. Here, we report characterization of mitogenome of *R. dominica* and its phylogenetic position. *Rhyzopertha dominica* complete mitochondrial genome (GenBank accession number MN527959) from Jingziguan town consisted of a circular DNA molecule of 15,862 bp (with 74.36% A + T content). The mitogenome comprised of 13 protein-coding genes (PCGs), and 22 tRNA and two rRNA genes. PCGs had typical ATN (Met) initiation codons and were terminated by typical TAN stop codons.

The lesser grain borer, *Rhyzopertha dominica* Fabricius is a primary pest of stored products containing starch in many regions of the world, which is the most difficult insect pests to control with insecticide grain protectants (Edde 2012). Here, we report the characterization of the complete mitogenome of *R. dominica* for molecular identification and phylogenetic studies.

Samples of adult *R. dominica* (GYU-20190630-002) were obtained from Jingziguan town (E 111.026°, N 33.244°), Xichuan county, Nanyang City, Henan province, China on 30 June 2019. Genomic DNA was isolated and fragmented to build a genomic library of Insert Size 400 bp that was sequenced (paired end 2 × 150 bp) using an Illumina HiSeq 4000. We obtained 26,180,130 reads of raw data and 25,075,888 reads of high-quality, clean data (95.78%). The genome was assembled *de novo* with A5-miseq v20150522 (https://github.com/koadman/docker-A5-miseq) (Coil et al. 2014) and SPAdes v3.9.0 (http://cab.spbu.ru/software/spades/) (Bankevich et al. 2012).

The mitogenome of *R. dominica* consists of a 15,862 bp circular DNA molecule, with 44.28% A, 30.08% T, 15.5% C, and 10.14% G, which has an A/T bias (74.36% A + T content). The AT- and GC-skews of the major strands of the mitogenome were calculated to be approximately 0.191 and –0.209, respectively. The length of the A/T-rich region in the mitogenome is 1452 bp, with 77.34% A + T content, and is located between the srRNA and tRNA-Ile.

The order and orientation of the functional areas of the *R. dominica* mitogenome are identical to those in the *Tenebrio obscurus*, *Zophobas atratus*, and *Blaps rynchopetera* mitogenome (Bai et al. 2018, 2019; Yang et al. 2019). The mitogenome of *R. dominica* contained 13 protein-coding genes (PCGs), and 22 tRNA and two rRNA genes. All 13 PCGs had typical ATN (Met) start codons and TAN stop codons; *nad2*, *cox1*, *cox2*, and *nad3* had ATA as a start codon, *atp8*, *nad5*, *nad6*, and *nad1* had ATT as a start codon, and *atp6*, *cox3*, *nad4*, *nad4l*, and *cob* had ATG as a start codon. From these, *nad2*, *atp8*, *atp6*, *nad4l*, and *nad6* had a TAA stop codon, *nad3*, *cob*, and *nad1* had a TAG stop codon, and *cox1*, *cox2*, *nad5*, *cox3*, and *nad4* had an incomplete stop codon consisting of a T, which was completed by the addition of 3′A nucleotides to the resulting mRNA. The 22 tRNA genes were interspersed throughout the coding region and ranged from 61 (tRNA-Cys) to 71 bp (tRNA-Lys) in length. lrRNA and srRNA were 1201 and 732 bp long, respectively.

To validate the phylogenetic position of *R. dominica*, the mitogenome DNA sequences from 16 species of Cucujiformia were used to construct a phylogenetic tree by the maximum likelihood method using the MEGA 7 software (Kumar et al. 2016) (Figure 1). *Rhyzopertha dominica* was closely clustered with *Dinoderus minutus*. In conclusion, our study provides information of the mitogenome of *R. dominica*, which will be useful for molecular identification and phylogenetic studies.

**Figure 1.**
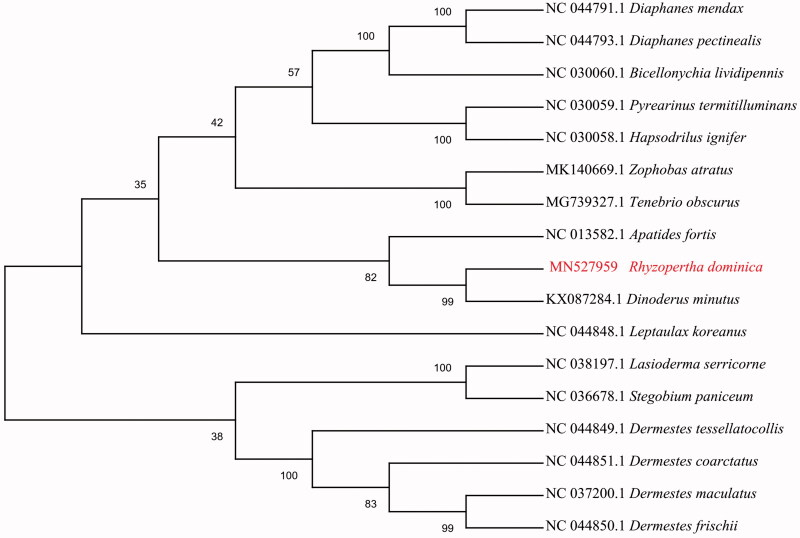
The maximum likelihood phylogenetic tree of *R. dominica* and other 16 beetles of Cucujiformia based on the DNA sequences of mitogenome.
